# Comparison of immunotherapy mediated by apoptotic bodies, microvesicles and exosomes: apoptotic bodies’ unique anti-inflammatory potential

**DOI:** 10.1186/s12967-023-04342-w

**Published:** 2023-07-17

**Authors:** Jing Wen, Dale Creaven, Xiangshu Luan, Jiemin Wang

**Affiliations:** 1 Department of Pharmacy, The Third Hospital of Changsha, Changsha, China; 2 Regenerative Medicine Institute (REMEDI), School of Medicine, College of Medicine, Nursing and Health Sciences, University of Galway, Galway, Ireland; 3 School of Life Science and Technology, China Pharmaceutical University, Nanjing, China

**Keywords:** Immunotherapy, Immunostimulatory, Immunomodulatory, Extracellular vesicles, Apoptotic bodies, Microvesicles

## Abstract

Immunotherapy, including immunostimulation and immunosuppression, has seen significant development in the last 10 years. Immunostimulation has been verified as effective in anti-cancer treatment, while immunosuppression is used in the treatment of autoimmune disease and inflammation. Currently, with the update of newly-invented simplified isolation methods and the findings of potent triggered immune responses, extracellular vesicle-based immunotherapy is very eye-catching. However, the research on three main types of extracellular vesicles, exosomes, microvesicles and apoptotic bodies, needs to be more balanced. These three subtypes share a certain level of similarity, and at the same time, they have their own properties caused by the different methods of biogensis. Herein, we summarized respectively the status of immunotherapy based on each kind of vesicle and discuss the possible involved mechanisms. In conclusion, we highlighted that the effect of the apoptotic body is clear and strong. Apoptotic bodies have an excellent potential in immunosuppressive and anti-inflammatory therapies .

## Introduction

Immunotherapy is divided into immunostimulation and immunosuppression according to the type of disease. Immunostimulation, also called immunostimulatory therapy, has shown excellent potential in treating cancer [1] and has also been explored in treating some viral infections [1]. Immunostumulation’s representative product is the PD-1 inhibitor to treat tumours. Targeting the suppression of the immune system is the aim of immunosuppressive/ immunomodulatory therapy. Immunomodulation is also widely used in anti-inflammatory treatment. This therapy is effective under several conditions: (i) after organ and/or tissue transplantation (graft-vs-host disease, GvHD); (ii) in autoimmunity; (iii) when it overreacts to allergens.

Extracellular vesicles (EVs) are widely studied. Types of EVs include exosomes, microvesicles (MVs) and apoptotic bodies (ApoBs) [2–6]. All three subtypes have therapeutic potential as they act as important messengers in physiological and pathological conditions. EVs all potentially and purposefully target immune cells to mediate immunotherapy.

**Fig. 1:**
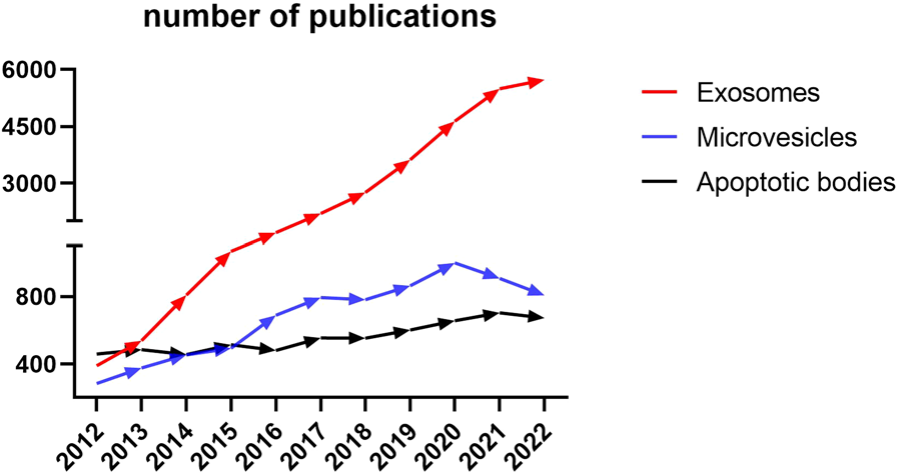
The number of publications about the three types of vesicles in the recent decade. The data was acquired by searching each vesicular name as “topic” in the webofscience.com. This figure was generated by GraphPad Software (9.0.0)

**Fig. 2:**
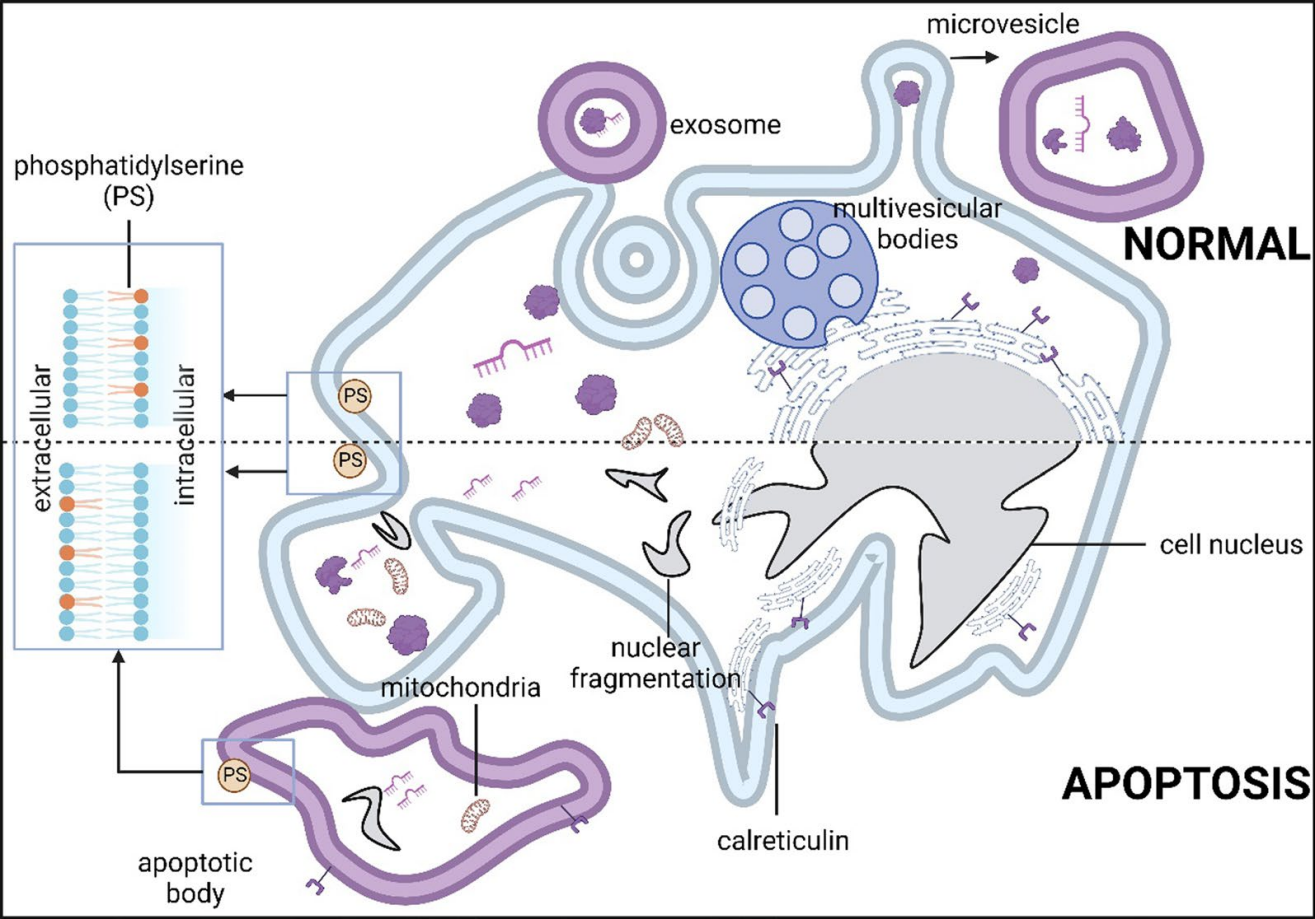
Biogenesis of three kinds of vesicles. Exosomes form by undergoing intracellular multivesicular bodies pathway. Microvesicles (MVs) are shed by outward blebbing of the plasma membrane. Apoptotic bodies (ApoBs) are produced by apoptotic cells. The process begins with condensation of the nuclear chromatin, followed by membrane blebbing, progressing to the disintegration of the cellular content into distinct membrane-enclosed vesicles

As one of the smallest types of EVs, exosomes range approximately from 30 to 150 nm [7–9]. Due to their therapeutic properties and delivery potential, exosomes have become an absolute research hotspot in the last decade (Fig. 1). Exosomes are small-sized particles formed during double invagination of the plasma membrane and the generation of intracellular multivesicular bodies (MVBs) wrapping intraluminal vesicles [10–12]. After MVB fuses with the plasma membrane, intraluminal vesicles are finally released through exocytosis as exosomes [12] (Fig. 2). Their small size (~ 50 nm) was considered to allow a higher cellular uptake than larger-size EVs in thermodynamic models and several experimental studies [7, 13, 14]. Although, its surface membrane protein CD47 can bind to SIRP-α to block phagocytosis by the immune cells [15, 16]. CD47 exempts the phagocyte system and enables them to target other immunocytes. Especially after modification/engineering, their target capacity can be significantly enhanced. Several studies have enabled exosomes to be involved in the phagocytes system. Artificial CD47 knock-out [17] and CD47/SIRP-α competitive occupancy [18] are two effective methods to allow exosomes to be phagocytosed.

**Table 1:** Simple comparison between exosomes, microvesicles (MVs) and apoptotic bodies (ApoBs)

	Size	Biogenic pathway	Origin	Major cargo
Exosomes	30–150 nm [7–9]	1. Endosomal sorting complex required for transport (ESCRT)-dependent pathway [19]; 2. Ceramide-dependent pathway [20]; 3. Tetraspanin pathway [21, 22]; 4. External stimuli [23, 24]	All cells	Nucleic acid (RNA), protein, and lipids [25]
MVs	100–1000 nm [26];	Direct outward budding [27]	All cells	Nucleic acid (RNA), protein, and lipids [26]
ApoBs	50-5000 nm [28]; 500–2000 nm [29]; 800–5000 nm [30]; About 1000–5000 nm [31]	Terminal stage of cell apoptosis [32]	Cells undergoing programmed cell death (apoptosis) [28]	Nucleic acid (DNA and RNA), protein, lipids, cell organelles and cell nuclear fragments [29, 33]

Microvesicles (MVs) are vesicles (0.1–1.0 μm) shed by outward blebbing of the plasma membrane [26] (Fig. 2). MVs shared similar properties with exosomes. MVs and exosomes are often merged and referred to as small EV (sEV). Both MVs and exosomes are multi-targeting. MVs and exosomes can target multiple cells depending on their parental cells. MVs also express vesicular CD47 [34, 35], they are speculated to escape phagocytosis in vivo and have a prolonged circulation time.

Depending on the different cell sources, MVs and exosomes can promote immunostimulation or immunosuppression. For example, MVs from tumours can present tumour antigens to the antigen-presenting cells (APCs) to mediate immunostimulation [36, 37], while mesenchymal stromal cell (MSC)-derived exosomes are immunomodulatory. MSC-exosomes have been shown to immunomodulate in many autoimmune diseases including, GvHD [38], rheumatoid arthritis [39] and multiple sclerosis [40].

Compared to exosomes and microvesicles, the enthusiasm to study ApoBs is lower (Fig. 1). However ApoBs have very similar properties to exosomes except for their larger size (50–5000 nm) [28]. They are produced by apoptotic cells. Apoptosis begins with the condensation of the nuclear chromatin, followed by membrane blebbing, progressing to the disintegration of the cellular content into distinct membrane-enclosed vesicles termed ApoBs or apoptosomes [29, 32] (Fig. 2). Like MVs and exosomes, ApoBs also strongly affect recipient cells, which are professional phagocytes and nonprofessional neighbouring cells [41]. But unlike MVs and exosomes, ApoBs’ target cells are less variable. Among all the recipient cells, the main target cells are macrophages, dendritic cells (DCs) and other neighbouring cells. Both macrophages and DCs play an important role in modulating the immune system. After phagocytosing ApoBs, macrophages are inclined to polarize to anti-inflammatory M2 phenotype [42] while, tumour-derived ApoBs induce DCs to pro-inflammatory mature phenotype [43].

Overall, exosomes, MVs and ApoBs have a similar lipid bilayer membrane and carry a gene andprotein cargo. Also, all of them are released by cells but through different pathways. They all can deliver their cargo or loaded drug to recipient cells and elicit a therapeutic effect. But in recent years, the enthusiasm to study ApoBs has been low despite their potent effect on immune cells. In this review, we stated the status of immunotherapy meditated by these three vesicles and with a specific interest in the the effect of ApoBs. The possible involved mechanism which causes the difference was also analyzed.

## Comparison of immunotherapy

### Immunotherapy mediated by exosomes

Exosomes’ recipient cells vary depending on the derivation. Homing effect to target their parental cells, ligand-receptor binding-mediated targeting and macrophage-dependent clearance are three major theories of exosome targeting. (1) The homing effect refers to the exosomes ability to home to their cells type of origin, for example, tumour-exosomes target and alter tumour cell in tumour mircoenviroment [44, 45]. (2) Ligand-receptor binding also called active targeting. Active targeting is where a targeting moiety, such as a ligand or an antibody, is introduced onto the exosomes to target tissues with specific upregulated proteins in comparison to the surrounding cells [46–48]. (3) Macrophage clearance is that exosomes are primarily cleared via phagocytosis and endocytosis by macrophages in the mononuclear phagocyte system (MPS) [49, 50]. These three targeting methods are compatible, and multiple mechanisms are often used together to design exosome treatment strategies.

#### Immunostimulation mediated by exosomes

The most typical immunostimulation model induced by exosomes is tumour immunotherapy elicited by tumour-derived exosomes. Regarded as a very potential tumour vaccine, exosomes carry sufficient antigens from their parent cells. After being presented by APCs or directly recognized by T-cell receptors [51], an immunostimulatory cascade reaction is initiated and thus leads to a beneficial pro-inflammatory anti-tumour effect [52].

However, the binding of exosomal surface CD47 and SIRP-α causes a “don’t eat me” signal [15, 53], enabling exosome’ immune escape from MPS. The low-efficient phagocytosis, resulting in less antigen-presenting, is a challenge of tumoral exosome immunostimulatory therapy. Through the blocking of CD47, the phagocytosis of exosomes by MPS increases but whether this loss of CD47 results in a stronger immunostimulatory reaction remains unknown.

To overcome this low-efficient phagocytosis obstacle, there are three main strategies.

1. In vitro incubating antigen-carried exosomes with DCs. It is verified that DCs are able to uptake exosomes in a simpler in vitro environment than in more complex in vivo environments [54]. In this paper, breast cancer cell E0771-derived exosomes were reported to contain immunomodulatory molecules such as HSP70, HSP90, MHC I and MHC II. After incubating with exosomes in vitro, mice dendritic cell DC2.4 cells increase the proliferation and migration abilities, accompanied by the upregulation of CD40 (a marker of mature DC). These DCs-treated tumour-bearing mice exhibited decreased tumour growth and sufficient T-cell infiltration [54]. Importantly, in another research paper, exosome-incubated DCs can induce stronger stimulatory reactions and anti-tumour effects than tumour lysate-incubated DCs [55], demonstrating the sufficiency and high efficiency of antigens carried by exosomes.
2. Using dying tumour cell-derived exosomes. DCs fail to recognize live tumour cells and cease to become activated, but DCs can be activated by antigens from apoptotic tumour cells. Thus, Zhou et al. prepared a dying tumour cell-derived exosomes to stimulate the immune system [56]. Although they did not quantify the apoptotic exosomal CD47, the uptake efficiency of apoptotic vesicles was very high, and the immunostimulation was also successfully triggered. The relevant mechanism of apoptotic vesicles likely involved the exposed phosphatidylserine, similar to ApoBs. This mechanism will be discussed in a later chapter.
3. Antigens, adjuvant or other therapeutic agent co-delivery. Generally, this co-delivery method is to strengthen immunostimulation. The research above using apoptotic cell-derived exosomes [56] also involves adjuvant and siRNA to enhance the therapeutic effect. Zhou et al. used MART-1 to expand T-cell-related responses and CCL22 siRNA to impede CCR4/CCL22 axis between Tregs and DCs. Commonly used adjuvant includes CpG DNA [57] and α-galactosylceramide [55].

Regarding, CD47-targeting strategy, exosomes can be utilized to block tumoral CD47 binding with MPS SIRPα, and thus leading to improved phagocytosis. This immunostimulation was caused by exosomes indirectly because exosomes are not regarded as the presented antigen. For example, by transfecting SIRPα plasmid DNA, SIRPα-expressed exosomes display an excellent affinity to CD47-naturally-overexpressed cancer HT29 cells. Due to the binding of tumoral CD47-exosomal SIRPα, macrophages cannot recognize tumoral CD47 “don’t eat me” signals and phagocytose more tumour cells. This will also enable intensive T-cell infiltration and thus reduce the volume of the tumour in vivo [16].

Except for tumour-derived cells/exosomes, M1 macrophage-derived exosomes are studied to enhance immunostimulation as M1 is a commonly-considered pro-inflammatory cell phenotype. Mice M1 macrophage cell RAW264.7-derived exosomes are reported to be able to increase M0 RAW264.7 releasing pro-inflammatory cytokines, while M2 RAW264.7-derived exosomes cannot. Exomsome treated M0 RAW264.7 secreted cytokines induce murine breast cancer cells 4T1 apoptosis [58].

#### Immunosupression mediated by exosomes

The previous paragraph introduced a lot of tumour-derived exosomes that cause immune stimulation. In fact, most of the tumour-derived exosomes naturally induce immunosuppression functions and are a very important component of the immunosuppressive tumour microenvironment [59–61]. These exosomes, especially PD-L1-expressing tumour exosomes, are produced in autologous tumour tissue and benefit tumour progression [62–64]. Autologous tumoral and PD-L1-carried exosomes contribute to immunosuppression and impede anti-PD-1 therapy [65] via inducing tumour-specific CD8 + T cell exhaustion [66] and suppression [67] and thus reducing immune infiltration. Blocking these immunosuppressive exosomes is a good strategy to overcome the low response of PD-L1 therapy. Using Macitentan to inhibit these autologous tumoral EVs secretion, the binding to PD-1 and PD-L1 decreases and thus enhancing the CD8 + T cell-mediated tumour killing and anti-PD-L1 therapy [68].

MSC have antigen-presenting properties [69]. This property is relevant to immunomodulation and immune tolerance [70–72]. Effecting similarly with their parental cells, MSC-exosomes are also reported to involve antigen-presenting pathways, which can induce more Tregs in the presence of DCs than absence [73]. This demonstrated that MSC-exosomes might present the relevant antigens to DCs, but not directly affect T cells. However, MSC-exosomes antigen presenting mechanism of action is currently lacking explanation. Despite the lack of explanation, the immunomodulatory effect of MSC-exosomes is widely verified. They can expand Tregs [73], polarize M2 macrophage [74] and inhibit T cell proliferation [75]. MSC-exosomes have potential in various autoimmunity diseases, including graft-versus-host disease [38, 76], rheumatoid arthritis [39, 77] and uveitis [78, 79].

### Immunotherapy mediated by MVs

MVs function similarly to exosomes. These two vesicles are often amalgamated and referred to as small EVs. Some studies compared the proteomics between exosomes and MVs [80, 81] to reveal the difference. It is likely that MVs carry more kinds of proteins than exosomes, possibly due to their larger size [82, 83]. MVs are more similar to their parental cells with more comparable protein categories than exosomes [83]. Although there are differences between these two particles, the conclusion is that both can reflect the state and function of their parental cells. No studies have shown a significant difference in function between these two particles. Additionally, MVs also express CD47 [34, 35] to enable extended circulation times and multi-targeting properties. In this review, we speculate MV and exosomes function similarly.

In the case of similar effects, tumour and MSC-derived MVs were also widely studied. Like their exosomes, tumour-derived MVs can induce mature DCs in vitro and cause an anti-tumour immunostimulatory response [37]. MSC-derived MVs are immunomodulatory and have demonstrated the ability to modulate inflammatory responses [84, 85]. Apart from tumour and MSC-MVs other immunotherapies of different MVs are also interesting. In the aspect of immunostimulation, activated CD4 + effector cells are immunostimulatory. Their MVs target microvascular endothelial cells. Proteomic analysis showed these inflammation-related cell-derived MVs were enriched with proteins involved in pro-inflammatory processes. CD4 + MVs have been shown to inhibit endothelial wound healing and induce endothelial cell apoptosis [86]. The activated T cell-derived MVs are also proven to deliver gene signal miR-4443 to the mast cell, leading to their activation in the T cell-mediated inflammation [87]. These facts further demonstrate MVs function similarly to their parental cells.

### Immunotherapy mediated by ApoBs

**Fig. 3:**
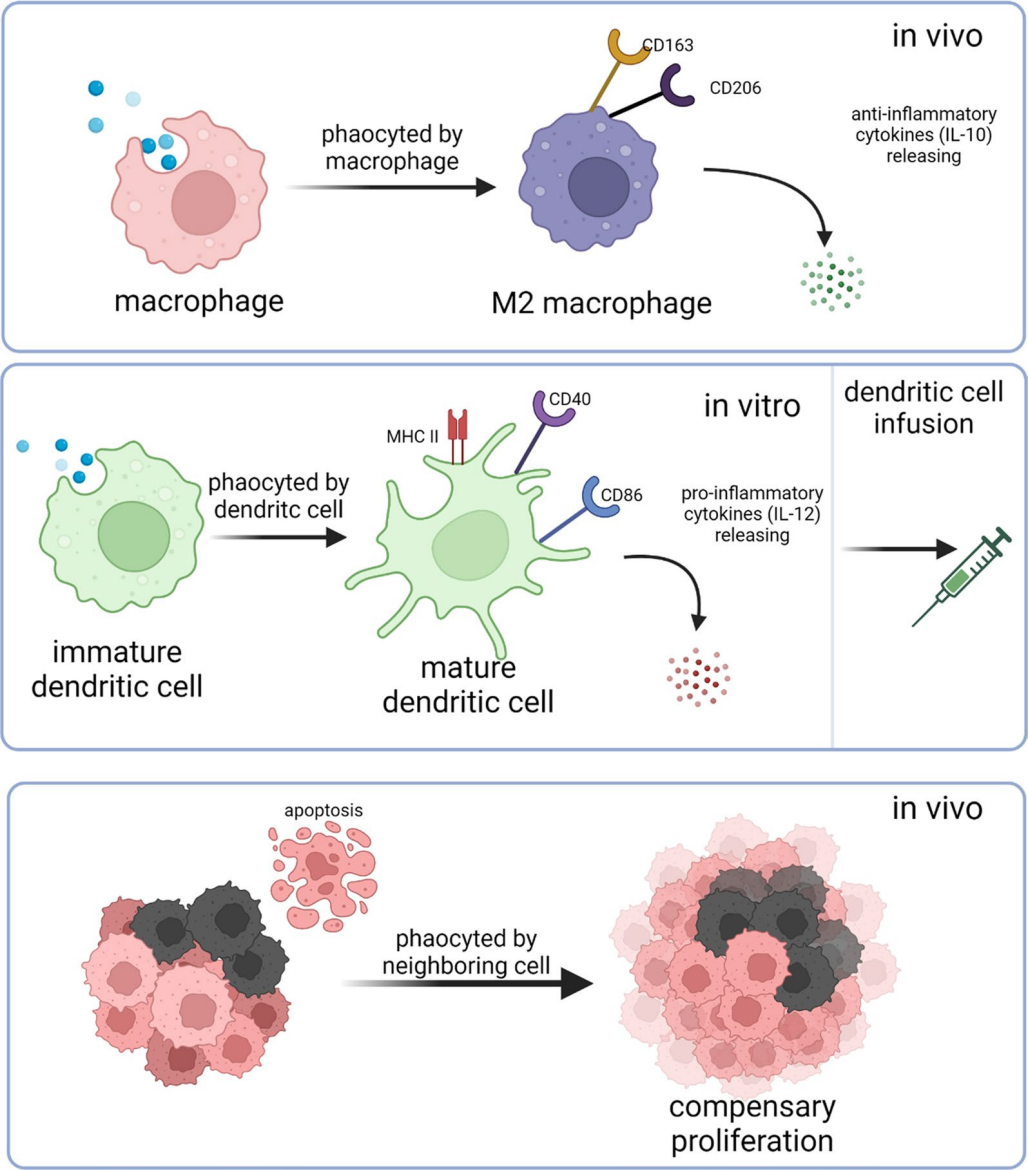
Three major pathways to clear apoptotic bodies. The clearance via macrophages and dendritic cells (DCs) can be used in immunotherapy

Unlike the multi-targeting of exosomes, ApoBs are eliminated by two main kinds of cells. The first is a “professional phagocyte” such as a macrophage and immature DC. The other is “nonprofessional neighbouring cells” such as the neighbouring tumour cells. More importantly, the lack of CD47 enables rapid clearance and is fewer off-target effects. Therefore, compared with EVs, the most potent advantage of ApoBs is a clear target cell (Fig. 3).

#### Anti-inflammatory and immunosuppressive: phagocyted by macrophages and inducing M2 phenotype

Macrophages are a category of white blood cell that engulfs and digests substances which do not have proteins specific to healthy cells on their surface. Macrophages are crucial in the initiation, maintenance, and resolution of inflammation. It is macrophages that eliminate ApoBs.

Furthermore, macrophages can induce host defense and inflammatory response or suppress these functions via phenotyping conversion [88–91]. The authors speculated that ApoBs or apoptotic cells contained “dead” and “injured” signals. The macrophages phagocyte ApoBs and initiate a negative feedback loop, in other words, marcophages differentiate into anti-inflammatory/ regeneration-facilitating M2 phenotype. Among all the ApoBs in mediating M2 macrophage, MSC-derived ApopBs are the most studied.

Recently, it has been reported that MSC undergo apoptosis before eliciting their functioning in vivo [92]. Then apoptotic MSCs and their efferocytosis-induced inflammatory pathways in alveolar macrophages mediate immunomodulation and reduce disease severity of autoimmunity [93]. The immunomodulatory potency of apoptotic MSCs is even higher than alive MSCs [94]. These findings likely reveal that apoptosis is the most immunomodulatory status of MSC. The eventual outcome of apoptosis is ApoBs production. This may predict the strong immunomodulation of MSC-derived ApoBs. In other words, MSC-ApoBs have the potential to be a more direct therapeutic agent than MSC itself. In addition, ApoBs are more in line with the concept of cell-free therapy, its immunogenicity is lower than MSC in theory. In all, MSC-ApoBs deserve a greater focus, equal to that of other EVs.

There have been some studies focusing on the immunomodulatory property of MSC-derived ApoBs via the mediation of macrophages. Liu et al. verified that MSC-ApoBs facilitate cutaneous wound healing by polarizing M2 macrophages [42]. Also, to induce M2 phenotype, Zheng et al. utilized MSC-ApoBs (apoptotic vesicles) to treat type 2 diabetes. They also showed efferocytosis of ApoBs can induce macrophages to reprogram transcriptionally in vitro and inhibit the infiltration and activation of diseased liver macrophages in vivo [95].

#### Pro-inflammatory: phagocyted by DCs

As mentioned above, macrophage phagocytosing results in an anti-inflammation response. This responses can switch to a pro-inflammatory status when the phagocytic cells become DCs. It has been widely reported in the establishment of apoptotic tumour cell-phagocytosed DCs for immunotherapy [96–100]. DCs that acquired antigens from apoptotic tumour cells are able to induce major histocompatibility complex (MHC) class I-restricted cytotoxic T cells and anti-tumor immunity [98]. This work inspired us to examine the role of ApoBs-phagocytosed DCs.

DCs, which load myeloma cell-derived ApoBs, can induce myeloma-specific T cells, leading to the activation of myeloma-reactive allogeneic T lymphocytes that produce IFN-γ [101]. Meanwhile, allogenic DCs from healthy donors, pulsed with leukemic cell-derived ApoBs, is a feasible and safe treatment for chronic lymphocytic leukaemia patients [43]. More importantly, compared with the lysate and the RNA from tumour cells, the ApoBs can induce a stronger autologous T-cell response in chronic lymphocytic leukaemia. In this clinical trial, ApoB-loaded DCs induce stronger T-cell responses with higher expression of IL-2 and IFN-γ [102]. This demonstrates ApoBs can induce better immunostimulatory DCs than tumour lysate. However, these ApoB-activated DC studies are slightly dated and have been completed more than ten years ago. Their method and technology to characterize ApoBs are limited. This is a clear limitation of these studies.

Compared to the macrophage-ApoBs strategy, the DC-involved ApoB study preferentially incubates ApoBs with DCs in vitro before injecting DC in vivo, while the ApoBs-macrophage strategy enables direct in-vivo fusion. We speculated this may account for (1) macrophage is the major population of tissue-resident mononuclear phagocytes [103]; (2) the increased phagocytosis capacity of macrophages [104]; (3) ApoBs are inclined to be phagocytosed by macrophages because of exposed phosphatidylserine (PS) [105]. If ApoBs can be accurately delivered to DC, infusing them in vitro prior to injection is unnecessary and the immunosuppression of tumour microenvironment will be greatly improved.

#### Phagocyted by neighbouring cells

The third way of phagocytosing ApoBs is through neighbouring cells. If the professional phagocyte is not abundant at the apoptosis site, nonprofessional neighbours usually clear ApoBs during development [106]. However, this phagocytosis does not directly connect to the immune response. In this neighbouring cell-phagocytosing mechanism, ApoBs mainly play a role in promoting cell growth and proliferation [107–110]. It is possible that macrophages and DCs can also grow or divide rapidly after phagocytosis of ApoBs.

### Modifiable (engineered) properties

**Fig. 4:**
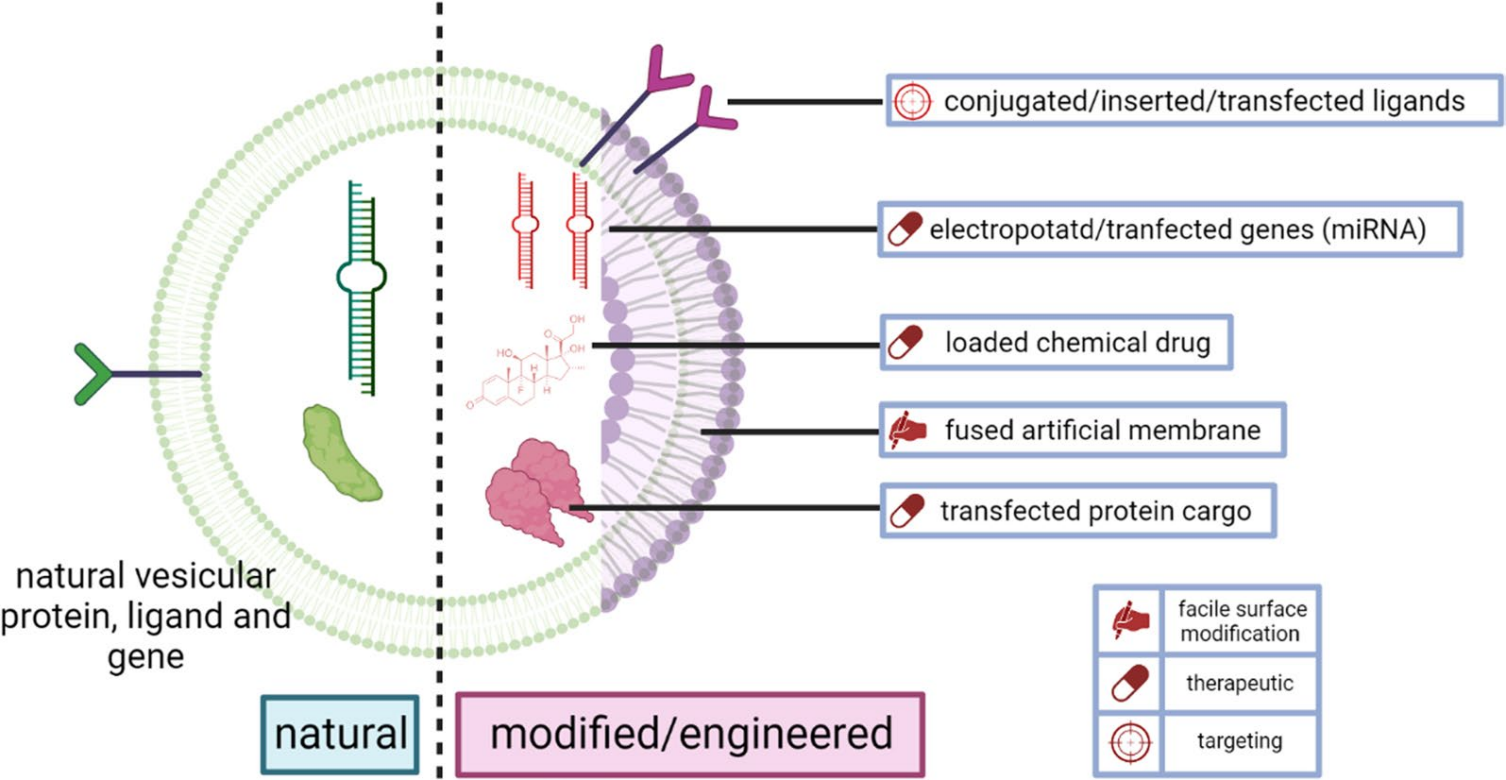
Commonly-used methods to modify the vesicles

Because exosomes, MVs and ApoBs all have a similar lipid bilayer membrane and a loading-feasible core, the modifiable property is shared by all these particles. In the above paragraph, we discussed some modified vesicles that illustrate a strengthened immunotherapy function. In brief, this modification can be separated into vesicle-core cargo loading, membrane modification and membrane fusion engineering (Fig. 4).

Firstly, natural loading is the most commonly utilized way to enhance the therapeutic efficacy on the basis that vesicles already load the natural therapeutical genes and proteins. Artificially loaded cargo implies altering the cargo by a variety of non-organic procedures and includes alterations to the genes and proteins. For example, 4T1 tumour cell-derived exosomes loaded miR-142, miR-155 or Let-7i respectively by electroporation [111]. Each kind of microRNA-loading enhances the effect on DC maturation and thus mediates an immunostimulatory response. On the contrary, monocytic THP-1 cell-derived exosomes loading miR-146a or miR494 exhibit an enhanced inhibitory effect on DC maturation [112]. These two studies sufficiently demonstrate the importance of vesicle cargo. These mircoRNA’s immunostimulatory or immunosuppressive effect needs to be verified in advance, and loading into exosomes amplifies their effect. As for protein delivery, ovalbumin is often loaded in the nanoparticle to mediate allergen-specific tolerance in the ovalbumin-induced allergic inflammation model. In an ovalbumin-caused allergic rhinitis mice model, exosomes play an important role as a messenger. Exosomes simultaneously deliver allergens (ovalbumin) and CpG DNA, an adjuvant that can induce a Th1 immune response, for the treatment of allergic rhinitis. Ovalbumin was expressed in the exosomes by transfecting ovalbumin plasmid DNA into cells. Ovalbumin-loaded exosomes were delivered to the mice’s nasopharynx-associated lymphoid tissue and were primarily absorbed by the DCs via intranasal administration. Intranasally administering ovalbumin-loaded exosomes increased ovalbumin-specific IgG antibody titers in vivo thus ameliorated the disease [113].

Beside genes and proteins, chemical drugs are also very popular to load into the vesicle. Due to their small molecular size, they are easy to load into EVs. For example, to reduce the associated multiple serious adverse effects of systematic dexamethasone (DEX) therapy and achieve an accurate delivery to inflamed kidney, DEX was encapsulated into macrophage-derived MVs, because macrophage-derived EV can interact with inflamed endothelium through exosomal adhesion molecules. Macrophage-derived MVs deliver DEX into the kidney and suppress renal fibrosis and inflammation without glucocorticoid adverse effects [114].

Secondly, membrane modification usually conjugates proteins or adaptors on the vesicle membrane. The conjugated protein (adaptor) has a high affinity to the specific protein. and it lead to an accurate delivery. For example, glioblastoma EVs, modified with a high-affinity ligand Lewis^Y^ by insertion, can target DC-specific intercellular adhesion molecule-3-grabbing non-integrin. This therfore potentiates EVs as anti-cancer immunotherapy [115].

In terms of membrane fusion, liposome membrane is often used to incorporate with vesicular membrane because of they share a similar bilayer structure. Liposome membranes are commonly used to enhance further the modifiable property of natural vesicles [116]. For example, Kang et al. fused liposomes with EVs to form hybrid vesicles. They further extrude these hybrid vesicles with superparamagnetic ferroferric oxide nanoparticles to achieve magnetism and then insert 1,2-distearoyl-sn-glycero-3-phosphoethanolamine-N-[dibenzocyclooctyl(polyethylene glycol) (DSPE-PEG-DBCO).The DBCO-combined nanoparticle can capture circulating melanoma cells by azide-DBCO recognition. Than the captured melanoma cells can be enriched by magnetism [117]. Generally, the EV membrane in this research plays a role in camouflage to escape the recognition by MPS. And liposome fusion is used to further conferred magnetism and lipid insertion. Seldom can EVs be modified complexly and directly like this.

## Involved mechanisms causing macrophagic post-phagocytic anti-inflammation of ApoBs

As demonstrated above, compared with the multi-target properties of MVs and exosomes, ApoBs’ target is much simpler, which tends to be uptake by the phagocyte system. This review summarized three main involved mechanisms of the target tendency.

### Different size: it is possible that larger ApoBs are more easily to be phagocyted by macrophages

Exosomes’ size is controversial. For decades, there has not been any accurate range for them, which is mainly because of the different methods for isolating and determining their size. It is now widely supported that exosomes are the smallest population of EVs. The size is approximately 30–150 nm [6, 9].

In this review, we exemplify liposomes, an analogy with EVs, to demonstrate the size-dependent property of EVs. Nowadays, on the one hand, direct evidence of EVs’ size-related superiority is lacking evidence. Liposomes and EVs both have a phospholipid bilayer membrane structure [118]. They were usually compared in functions.

The small size is of advantage. First of all, a smaller size signifies high oral bioavailability. (Although there is little to no oral EV immunotherapy, milk-derived EV has been studied for oral administration [119].) Ong et al. [120] used griseofulvin as a model drug encapsulated by different sizes of liposomes. Smaller liposomes’ (≤ 400 nm) bioavailability was higher by approximately three times compared to larger liposomes (≥ 400 nm). Meanwhile, the smaller size also signifies improved stability, Farooq et al. [121] modified liposomes with D-α-tocopheryl polyethylene glycol 1000 succinate (TPGS) to generate TPGS-liposomes of smaller sizes. As a result, after 7 days at 4 °C, the size increment of the TPGS-liposome was less than the common liposome. And after 28 days, the modified smaller liposome still could keep higher encapsulation efficiency than the large more, common liposome.

Compared to exosomes, ApoBs have a wider range, which spans from 50 to 1000 nm. It is mainly because ApoBsares lysed from apoptotic cells. So its size is uneven. According to the current literature most of ApoBs are about 1 μm [30]. The Large size may represent targeting macrophages because phagocytosis is regarded as the uptake of particles larger than 0.5 μm [122]. This theory was also proven in recent years. For example, 1000 nm-liposomes could deliver the drug to rat alveolar macrophages better than smaller liposomes [123]. Only liposomes larger than 600 nm can lead to mononuclear phagocytes secreting IL-2 to induce the Th1 immune response [124].

### Different surface molecules: ApoBs have “eat-me” signatures

**Fig. 5:**
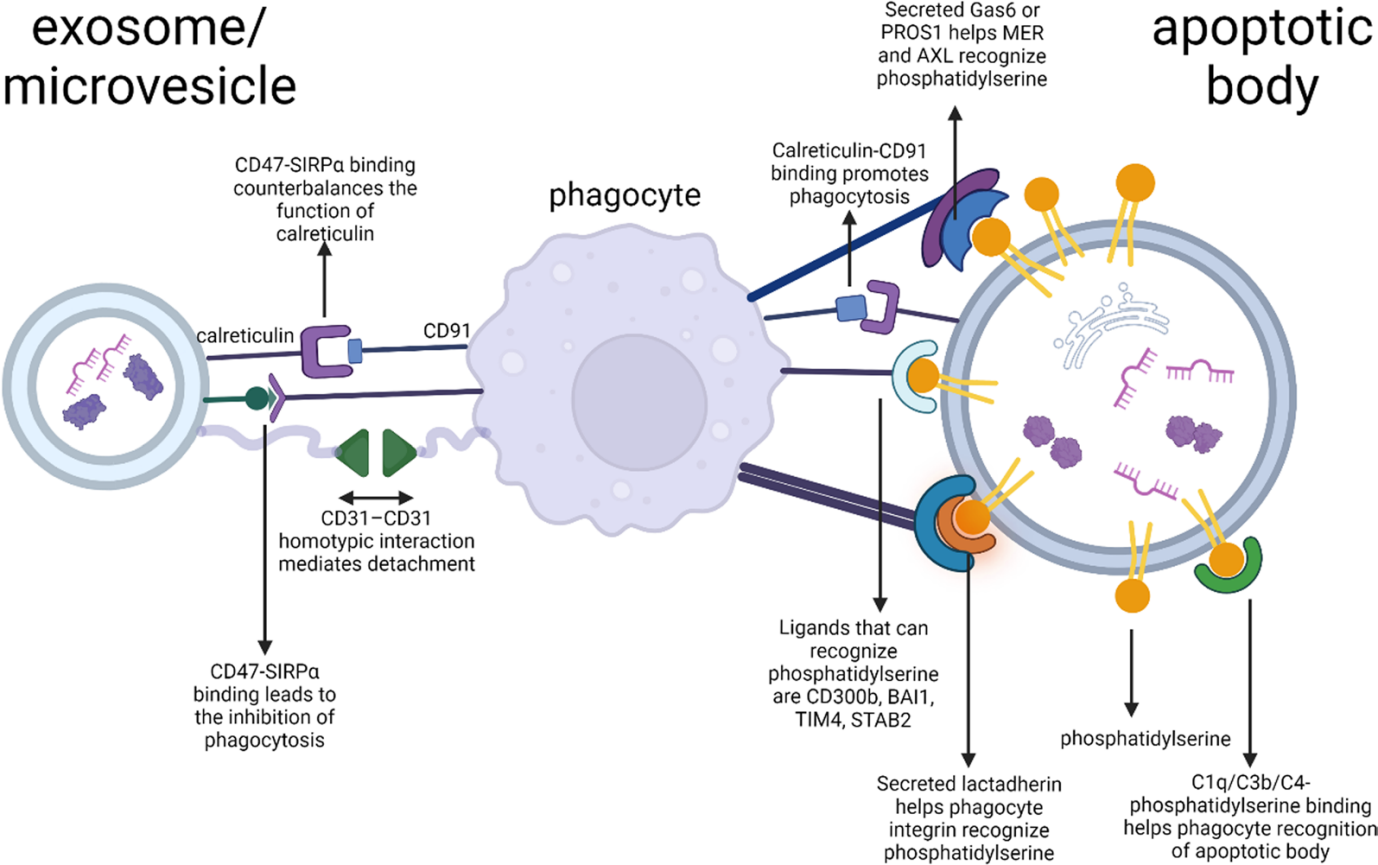
The molecules involved in the phagocytosis of ABs by macrophages

Apoptotic cells and ApoBs are inclined to be phagocyted by macrophages. They can be recognized by a variety of receptors on the surface of macrophages that can bind to apoptotic surface ligands and phagocytosis is initiated. Apoptotic cell surfaces are characterized by decreased “don’t eat me” molecules such as CD47 [125, 126] and CD31 [127–129], and overexpressed “eat-me” signals, such as cell-surface calreticulin and exposed phosphatidylserine (Table 1; Fig. 5). These alterations trigger phagocytosis via macrophages, which then drives polarization to M2 phenotypes and anti-inflammatory signalling (Table 2).

**Table 2:** The molecules involved in the phagocytosis of ApoBs by macrophages

Ligand	Presence or Function			Category	Signal	Receptor	References
	ApoBs	MVs	exosomes				
CD47	×	√	√	Protein	“don’t eat me”	SIRP-α	[17, 34, 35, 130, 131]
CD31	×	√	√	Protein	“don’t eat me”	CD31	[127, 129, 132–134]
Calreticulin (CRT)	√	×	×	Lipid	“eat-me”	CD91 (LRP1)	[95]
PS	√	×	×	Lipid	“eat-me”	C1q, C3b and C4; CD300b; BAI1; TIM4; Gas6/PROS1; STAB2; Lactadherin	[94, 135, 136]

Among these signals, the exposed PS is the most widely-studied “eat-me” signal [94]. When apoptosis occurs, PS will move from the inner leaflet of the plasma membrane to the outer leaflet. By binding to the surface of phagocytes, after phagocyte recognition and engulfment, anti-inflammatory signaling is triggered within these phagocytes.

By utilizing PS-mediated “eat-me” signal, there are several ApoB-inspired nanoparticles prepared: (1) Utilizing macrophage’s affinity to PS, Liu et al. [137] synthesized ApoB-mimicking nanoparticles. This nanoparticle could externalize its inner PS triggered by overexpressed matrix metalloproteinase 2 (MMP2) in the tumour site. Once phagocytosed by macrophages, it releases cytotoxic drugs to kill the macrophages and then damage the tumour microenvironment. (2) Using PS, Kraynak et al. [138] mimicked ApoBs by presenting it in the context of a generic stromal cell membrane from 3T3 fibroblasts. The PS-supplemented particle is anti-inflammatory without the use of any other drugs. Moreover, they verified PS incorporation not only improves relative uptake by macrophages but also polarizes the marcophages to their M2 phenotype.

Aside from for PS, calreticulin (CRT), localized normally in the endoplasmic reticulum lumen, is also transferred to the outer cell membrane where, along with PS, facilitates phagocytosis [139].

### The possible essence of phagocyted ApoBs: anti-inflammation

As mentioned above, EVs play a similar role to their parental cells. In this situation, ApoBs to some degree, function more similarly to their apoptotic parental cells rather than live cells. Apoptotic cells in vivo are more on the anti-inflammatory side. Apoptosis, which occurs every day, is regarded as an important way to maintain homeostasis [140, 141]. Apoptosis is also known as a significant death pathway and can trigger new cell development. Contrary to apoptosis, histiocytosis is characterized as pro-inflammatory [142–145].

In the above chapter, DCs phagocyte ApoBs and then trigger the immunostimulatory response. However, this reaction needs to be intervened in vitro. In other words, naturally occurring apoptosis and phagocytosis triggers anti-inflammation responses. The phagocytosis process of apoptotic material also promotes intrinsic mechanisms such as tissue growth and remodeling, regeneration and resolution of injury and inflammation.

In terms of the apoptosis-involved anti-inflammatory mechanism, at first, apoptosis, especially caspase-dependent apoptosis, is an immunologically silent form of cell death [146]. Caspases regulates inflammation by acting on two opposing functions. By catalyzing pro-inflammatory cytokine production, “inflammatory” caspases trigger inflammation [147]. On the contrary, “apoptotic” caspases safeguard against the triggering of inflammation by imposing a cell-death form that withholds the release of alarmins by dying cells and dictates the generation of anti-inflammatory mediators [148]. A recent study also demonstrated the inducible caspase-9-mediated apoptotic MSC exhibited stronger immunosuppressive properties than conventional MSC in vitro [149]. Besides, anti-inflammatory caspase, exposed-PS, a typical signal on the surface of the apoptotic cells, is a global immunosuppressive [150] and anti-inflammatory [151] molecule. PS-dependent ingestion of apoptotic cells promotes macrophage-secreting TGF-β1 and the resolution of inflammation [152, 153]. Also, in tumours, PS released from tumour apoptotic cells polarizes M2-like macrophage via the PSR-STAT3-JMJD3 axis [135]. Based on this strong polarized effect by PS, in recent years, there have been studies using PS liposomes to induce the M2 macrophage phenotype [154–156].

In addition, the post-phagocytosis mechanism of the apoptotic cells has also been summarized as (1) Nuclear receptors actively inhibit the formation of pro-inflammatory cytokines; and (2) Macrophages respond to the uptake of apoptotic cells/ ApoBs by releasing anti-inflammatory cytokines [146].

## Conclusion

To conclude exosomes, MVs and ApoBs have similar structures and modifiable properties. The effect of exosomes and MVs vary more, ranging from immunostimulation to immunosuppression, depending on the parental cell. But ApoBs in vivo mainly exert immunosuppressive functions. It is very clear that ApoBs target professional and non-professional phagocytes. Based on their immunosuppressive and anti-inflammatory properties, ApoBs are likely to have big potential in treating autoimmunity and inflammation.

## Abbreviations

ApoB: Apoptotic body
APC: Antigen-presenting cell
CRT: Calreticulin
EV: Extracellular vesicle
DC: Dendritic cell
DEX: Dexamethasone
GVHD: Graft-vs-host disease
MMP2: Matrix metalloproteinase 2
MHC: Major histocompatibility complex
MSC: Mesenchymal stromal cell
MV: Microvesicle
MPS: Mononuclear phagocyte system
MVB: Multivesicular body
PS: Phosphatidylserine
